# A Taxonomy of Non-honesty in Public Health Communication

**DOI:** 10.1093/phe/phad003

**Published:** 2023-03-23

**Authors:** Rebecca C H Brown, Mícheál de Barra

**Affiliations:** Oxford Uehiro Centre for Practical Ethics, University of Oxford, Suite 8 Littlegate House, 16-17 St Ebbe’s Street, Oxford OX1 1PT, UK; Centre for Culture and Evolution, Brunel University London, Uxbridge, England

## Abstract

This paper discusses the ethics of public health communication. We argue that a number of commonplace tools of public health communication risk qualifying as non-honest and question whether or not using such tools is ethically justified. First, we introduce the concept of honesty and suggest some reasons for thinking it is morally desirable. We then describe a number of common ways in which public health communication presents information about health-promoting interventions. These include the omission of information about the magnitude of benefits people can expect from health-promoting interventions, and failure to report uncertainty associated with the outcomes of interventions. Next we outline some forms of behaviour which are generally recognised by philosophers as being non-honest, including deception, manipulation, and so on. Finally, we suggest that many of the public health communicative practices identified earlier share features with the non-honest behaviours described and suggest this warrants reflection upon whether such non-honesty is justified by the goals of public health communication.

## Introduction

Communication is a cornerstone of public health activities. Yet there has been surprisingly little attention paid to the ethics of public health communicative practice. This may be due to a widespread assumption that attempts to change behaviour through information provision (as opposed to more intrusive means such as coercive regulation, or unobserved means such as nudges) are benign. There are, however, ways in which public health communication may fail to meet ethical standards. During the COVID-19 pandemic, for example, questions arose as to whether some recommendations were intentionally misleading: Anthony Fauci, the chief medical advisor to the US president, suggested that the vaccination herd immunity threshold was around 60–70%. He later claimed that this did not, exactly, reflect the truth as he understood it at the time. Rather, such statements were intended to influence behaviour at the cost of honesty (McNeil Jr., 2020; Prasad, 2020; Tufekci, 2020). The particular concern here is not that Fauci and other public health leaders had a mistaken understanding of the science of COVID-19, but that they strategically misrepresented their beliefs in communications to the public.

In this paper, we shall consider one aspect of the ethics of public health communication: whether or not common public health communicative practices can be considered honest. We will not make a case for the all-things-considered desirability of honesty, but will assume that, all else being equal, it is preferable that public health communications are honest. With this in mind, it is worth considering whether or not public health communications routinely fall short of honesty.

Elsewhere (de Barra and Brown, 2023) we attempt a more systematic discussion of the frequency with which public health communications fail to meet some basic requirements of honesty. Here our intention is not to quantify failures of honesty, but to illustrate some of the ways in which communication may fall short of honesty. In the section *Honesty as a Virtue* we provide some background to the virtue of honesty. In the section *Public Health Communicative Practices*, we identify a number of public health communicative practices which we think may, at least some of the time, be inconsistent with honesty. In the section *Forms of Non-honesty* we introduce, in general philosophical terms, a number of non-honest behaviours before, in the section *Are Such Communicative Practices Honest?*, discussing whether some of these feature in the public health communicative tools previously identified.

## Honesty as a Virtue

Honesty has been described by Miller (2021) as involving ‘reliably not intentionally distorting the facts as the agent sees them’. Adopting an Aristotelian approach, Miller takes honesty to have a corresponding vice of dishonesty. It is reasonably challenging to meet the demands of honesty, and it is possible for a person to fall short of honesty (by, for instance, failing to be motivated to act as honesty requires, for the right kinds of reasons) without being of dishonest character. So agents may qualify as neither honest nor dishonest, and instead occupy some middle ground of being ‘non-honest’. When evaluating actions, we might extrapolate from Miller’s analysis to describe those actions aimed at ‘not intentionally distorting the facts as the agent sees them’ as being consistent with honesty, and those which fail to aim at intentional fact non-distortion (either through active attempts to distort the facts, or through a lack of concern to avoid distortion) as being inconsistent with honesty, and at least some of the time consistent with dishonesty. For short-hand, we might describe the former as ‘honest actions’ and the latter as ‘non-honest’ actions.

Honesty is generally thought desirable, if not obligatory. Important exceptions may arise where the consequences of honest actions are significantly worse than their dishonest counterparts. Kant infamously insists that even when a murderer is at the door one is not permitted to lie, even if telling the truth will foreseeably result in an innocent person being killed (Kant, 1786/2012). Such ‘rigourism’ has turned many away from Kantian deontology, and resulted in a search for ways in which Kantian theory can consistently hold that it *is* permissible to lie under such circumstances.

The reasons for valuing honesty might be varied. These include outcome-based reasons, which Miller lists as potentially including the promotion of: rational decisions, actions and relationships; well-being and flourishing; respect for others and autonomy; harm avoidance; trust, trustworthiness and credibility; interpersonal coordination; and justice (Miller, 2021). Alternatively (or in addition), honest motivation might itself be intrinsically good, such that performing actions from an honest motivation will be good independent of the consequences.^1^ In discussing the honesty of public health communication, we will focus on whether or not communication appears to distort the facts,^2^ setting aside questions of motivation insofar as these also determine whether or not an action (or the character of an agent) should count as honest.

## Public Health Communicative Practices

We have suggested there are *prima facie* reasons for public heath communication to be honest, although the extent to which honesty (and other norms) should have a role to play in scientific communication is disputed (see in particular (John, 2018); see also (Brown *et al.*, 2022)). For now, we assume that, all else being equal, honest forms of communication will be preferable to non-honest forms, and that reflecting upon the honesty (or otherwise) of common public health communicative practices will be worthwhile. We shall now consider some such practices that might involve non-honesty or dishonesty. These are summarised in Table 1.

**Table 1.** Common potentially non-honest public health communicative practices

Imprecision and omission of decision-relevant information
Magnitude neglect
Harm neglect
Denominator neglect
Information of no or minimal relevance
Outcome swamping
Relative over absolute risk presentation
Mismatched reporting
Upper bounds rather than central estimates
Misrepresenting scientific justification
Spurious precision
Uncertainty laundering
Causation laundering
Eliding absence of evidence with evidence of absence
Bogus legitimisation

### Magnitude Neglect

Public health information often fails to indicate the effect size of the benefits and harms of health behaviours or interventions. For instance, the NHS website provides information about cervical screening including what it involves, why it’s important, who’s invited, and so on. It says: ‘Try not to put off cervical screening. It’s one of the best ways to protect yourself from cervical cancer’. There is no information on the NHS webpage about how likely one is to benefit from attending screening (e.g. the probability that one will die from cervical cancer if one regularly attends screening versus if one does not attend).

Elsewhere (de Barra and Brown, 2023) we analysed information provided on a range of public health websites, including (inter)governmental organisations such as the NHS, World Health Organization (WHO) and CDC; research charities such as Cancer Research UK (CRUK); and media companies such as Healthline and WebMD. Consistent with previous research that looked at specific health behaviours or a narrower set of information providers, we found that the benefits of health promoting interventions such as weight loss, healthy diet, physical activity, smoking cessation and reduced alcohol consumption were typically described qualitatively, with no quantification of the expected benefits and harms.

### Harm Neglect

Changes to behaviour and medical interventions typically involve costs and (risk of) harms. These can be medical, as in the case of overdiagnosis and overtreatment resulting from screening programs, as well as psychological, financial or time-based. In many cases, costs and harms will be reasonably transparent: one can easily imagine how changes to diet or wearing a mask will impinge on one’s wellbeing. But where the costs and harms are more difficult to intuit (perhaps because they are probabilistic rather than certain, or long-term rather than immediate) we might expect health communicators to be clear about these harms even if it reduces uptake of the relevant behaviour. The NHS cervical screening website, mentioned above, does list some potential harms of screening – temporary light bleeding/infection, ‘treating cells that may have gone back to normal on their own’, and early delivery during pregnancy (noted as rare). Commonly, public health communications fail to describe harms or present harms in a less prominent way than the benefits, with little detail given, including no quantifiable information regarding how frequently these harms occur. In *mismatched reporting* below, we further discuss asymmetric presentation of harms and benefits.

### Denominator Neglect

Communicators sometimes provide information about how common a condition is or how many lives are saved through an intervention. In the absence of other contextual data, these do not communicate anything about an individual’s risk of experiencing a particular disease, nor the likelihood that she will benefit from engaging with health promoting interventions. For example, the only quantification of benefit on the NHS page about breast screening states that ‘[b]reast screening saves around 1300 lives each year in the UK’ (NHS, 2021b). While this may be somewhat interesting, it does not tell the reader what proportion of women benefited from screening. In some circumstances, it may be perfectly reasonable to use raw numbers like these: health communication may be directed towards policy makers or members of the public interested in understanding the relative importance of different screening programs, for example. However, for individuals making decisions about how to allocate their efforts and precautionary behaviour, the absence of a denominator can render the numbers uninformative or misleading.

### Outcome Swamping

Public health communication often lists numerous benefits (and, less frequently, harms) of health promoting behaviours. For instance, the Mayo Clinic web page on obesity lists the following ‘complications’: heart disease and strokes, type 2 diabetes, cancer (including cancer of the uterus, cervix, endometrium, ovary, breast, colon, rectum, esophagus, liver, gallbladder, pancreas, kidney and prostate), digestive problems (including heartburn, gallbladder disease, and liver problems), sleep apnea, osteoarthritis, severe COVID-19 symptoms. They further describe how obesity can ‘diminish the overall quality of life’ by stopping you doing physical activities, causing people to avoid public places, discrimination, depression, disability, shame and guilt, social isolation, and lower work achievement (Mayo Clinic, 2022). There is no explicit indication given of how likely any of these outcomes are for someone who is obese, nor the degree to which attempting to lose weight is expected to reduce the risk of these outcomes.

### Relative Over Absolute Risk Presentation

Although numerical estimates of the magnitude of interventions’ effects are often absent, where they are provided they are often presented in a way that makes small effects appear large (Akl *et al.*, 2011; Caverly *et al.*, 2016). This is because communicators often present effects in terms of relative risk, rather than absolute risk or frequency data. Reporting that ‘mammography screening reduces the risk of dying from breast cancer by 25%’ might make mammography screening sound very effective, whilst the same data, reported in terms of frequencies – ‘1 less woman out of a 1000 will die of the disease’ – sounds somewhat less impressive (Gigerenzer *et al.*, 2007). Relative risks are opaque, insofar as they tell you nothing about what your risk of attaining some benefit (or suffering some harm) actually is – they only tell you how an intervention, behaviour, or condition will change that risk. It is more common for health information to be presented in the form of relative risks than in the form of absolute risks or frequencies, which are a more transparent way of describing risks. It has been argued that reporting absolute outcomes is less likely to promote certain cognitive biases and better support decision-making between interventions (Sedrakyan and Shih, 2007; Caverly *et al.*, 2016; Sprenger and Stegenga, 2017).

### Mismatched Reporting

Sometimes the reporting of relative risks is combined with absolute risks. Mismatched reporting involves describing benefits in terms of relative risks and harms in terms of absolute risks or frequencies. The result is to simultaneously exaggerate the benefits and minimise the harms of an intervention (or risks of non-intervention). For instance, Wegwarth and colleagues describe how the US Preventative Services Task Force uses mismatched statistics to report the benefits and harms of screening. The Task Force reports that ‘Sigmoidoscopy screening reduced the risk of death by 59%’ and that ‘Perforations are reported to occur in approximately 1 of 1000–10,000 rigid sigmoidoscopic examinations’ (Wegwarth and Gigerenzer, 2011). Caverly and colleagues (2016) also found that cancer screening guidelines used mismatched reporting to describe benefits and harms of screening about half the time. Harms are typically described as ‘risks’, emphasising their probabilistic nature whereas benefits are described in more deterministic terms. Indeed, when harm information is provided, it is more likely to be accompanied by qualifiers of uncertainty (de Barra and Brown, 2023) (see *Uncertainty laundering* below).

### Upper bounds rather than central or range estimates

The NHS website describes how exercise ‘can reduce your risk of major illnesses, such as coronary heart disease, stroke, type 2 diabetes and cancer and lower your risk of early death by *up to 30%*’ (NHS, 2021a) (emphasis added). Here, the NHS presents the risk reducing effects of exercise in terms of the most optimistic – and, perhaps, least likely – figure. Presenting quantitative information in terms of the upper or lower bounds of the likely effect size is common, occurring in around a fifth of claims from public health websites analysed (de Barra and Brown, 2023). Presenting quantitative risk information in terms of central estimates or ranges is more likely to be relevant to the average reader.

### Spurious Precision

Public health communication can sometimes present information in a way that suggests more precision than is warranted. John (2022) provides an account of spurious precision in health communication, including things like the 5 A Day campaign to encourage people to eat (at least) five portions of fruit and vegetables a day; BMI (body mass index) categories; a specific blood pressure threshold indicating hypertension, and so on. John argues that what marks a number out as being spuriously precise is that it could be replaced with a different but *equally justifiable* number. Indeed, regarding the 5 A Day recommendation, different countries have taken the same WHO report and interpreted it to mean that people should eat 7–8 portions for women/9–10 for men (Canada); 5 × 200g portions (the UK portion size is assumed to be 80g) (Austria); and 2 + 2 portions (an equal split of fruit and veg) (Singapore) (Peters, 2014; John, 2022). Spurious precision might obscure the fact that there is no single, non-arbitrary way of delineating healthy from unhealthy vegetable consumption, BMI, or blood pressure. Instead, there is some more complicated relationship between these markers and health. Spurious precision can also mask significant uncertainties in the value of therapies (see *Uncertainty laundering* below).

### Uncertainty Laundering

Public health communications rarely stress uncertainty. Yet uncertainty is inherent in the scientific evidence base which underlies claims about the health effects of a behaviour or intervention. First, since effects of health interventions are probabilistic, there is uncertainty over who will benefit and who will not. This uncertainty is usefully conveyed in number needed to treat (NNT) statistics, which express the number of people who would need to change their behaviour in order for one person to experience a health benefit. Second, researchers can also be uncertain about the proportion of people that can expect benefit. Consider how the NHS website describes the beneficial effects of exercise on happiness and depression:

Exercise is the miracle cure we’ve always had, but for too long we’ve neglected to take our recommended dose. Our health is now suffering as a consequence. […] This is no snake oil. Whatever your age, there’s strong scientific evidence that being physically active can help you lead a healthier and happier life. […] Given the overwhelming evidence, it seems obvious that we should all be physically active. It’s essential if you want to live a healthy and fulfilling life into old age. […] It’s medically proven that people who do regular physical activity have lower risk of […] depression.

(NHS 2021a)

One would expect such claims to be based on systematic reviews of intervention studies, like for example, Gordon *et al.*’s (2018)  *JAMA Psychiatry* meta-analysis. This paper finds that the NNT for exercise was about four, indicating that for every four people who undergo an exercise intervention, one will experience some meaningful improvement in depression scores whereas three will not. Although there may be drawbacks with using NNT as a metric for evaluating changes on a continuum, this still suggests that only a subset of people will benefit in a meaningful way from exercise. Readers may misinterpret the language of certainty (‘miracle cure’, ‘overwhelming evidence’, ‘medically proven’) to mean that the effects of exercise are guaranteed (everyone experiences a benefit). Further, the likely extent of that benefit is not mentioned – an example of *Magnitude Neglect* discussed above.

The exercise systematic review described above found that the effects of exercise were about half as large when the experimenter did not know whether the participant had been in the exercise or control group when they measured participants’ depression. The fact that less biased (but by no means unbiased) studies show much smaller effects should weaken our confidence about the effects of exercise on depression.^3^ While we might not expect the NHS page on exercise to cover technical issues of trial design, there appears to be a meaningful difference between the tentative nature of the scientific conclusions and the more strident claims made by the website. As we argue elsewhere (Brown *et al.*, 2022), there are good ethical and practical reasons for being frank about scientific uncertainty and the possibility that the research community’s best guess about the benefits of interventions will likely change over time.

### Causation Laundering

The WHO states that ‘Breastfeeding improves IQ […]’ (WHO, 2021). This neglects significant uncertainty in the scientific community about how to account for important confounds like socioeconomic status and parental intelligence when estimating the effects of feeding practices on IQ. Studies that attempt to isolate the effects of breastfeeding from these different factors have produced very different estimates for the effects of breastfeeding on IQ with some of the stronger designs showing no effect whatsoever (Yang *et al.*, 2018; Pereyra-Elías *et al.*, 2022). We term this *causation laundering*: it involves diluting or abandoning uncertainty about the causal nature of an association between some exposure and a health outcome, typically when claims of causation are drawn from research that is ill-equipped for drawing causal inferences (e.g. simple correlational studies rather than randomised controlled trials, instrumental variable approaches, or triangulation from multiple study types).

Note that causation laundering is often implicit rather than explicit. Under Gricean communicative norms,^4^ the statement that ‘there is a strong association between’ a behaviour and a health outcome can reasonably be interpreted as a causal claim by a lay audience: why else would the association be highlighted if not to provide people with some useful information about how to act on their environment in order to achieve that desired outcome? (Grice, 1975)

### Eliding Absence of Evidence With Evidence of Absence

Sometimes it is necessary to communicate that interventions are ineffective or that a particular behaviour is unlikely to make a difference to health. Public health communicators sometimes use ambiguous language when describing the lack of evidence for effectiveness. For instance, in April 2020 the WHO put out a statement that ‘There is currently no evidence that people who have recovered from COVID-19 and have antibodies are protected from a second infection’. (WHO, 2020) This statement was in response to suggestions that immunity passports might be issued to those who had been infected and recovered from COVID-19. The statement can be read as suggesting different things: ‘scientists have looked for evidence of COVID-19 immunity and cannot find it’ (i.e. there is probably no immunity to COVID-19) or ‘scientists haven’t looked for evidence of COVID-19 immunity yet, so haven’t found any’ (i.e. there might be immunity, but we don’t know yet). This ambiguity allows the WHO to suggest a pessimistic outlook on the likelihood of COVID-19 immunity (and thus the non-viability of immunity passports), whilst stopping short of explicitly making any predictions about the likelihood of immunity.

### Bogus Legitimisation

**Figure 1.**
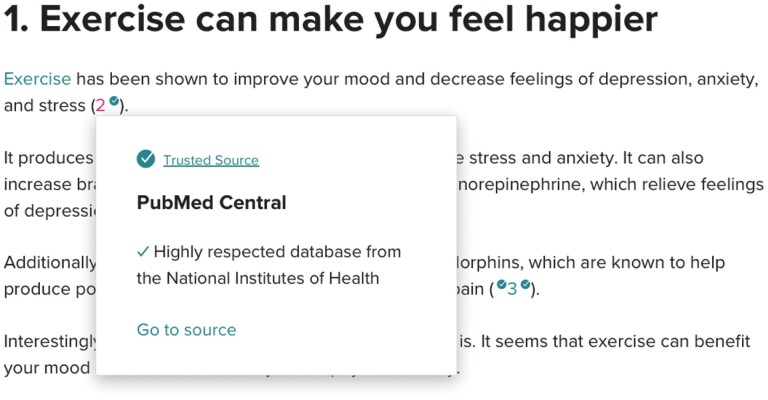
Screenshot from healthline.com.

Public health communicators might look to assert their legitimacy in various ways, some of which might be superficially credible, but in fact meaningless. For instance, Healthline, a popular health information website, provides links to sources they cite to support health claims (see Figure 1) (Healthline, 2021). These links are typically to articles stored on PubMed Central, an online repository of free-to-access biomedical publications. Healthline describes PubMed Central as a ‘Trusted Source’ and a ‘Highly respected database from the National Institutes of Health’ which might lead readers to assume that any information accessed through PubMed Central is backed by the NIH. Yet the quality of publications will vary and the mere existence of an indexed publication making a claim provides scant evidence that the claim is true. Moreover some ‘predatory journals’ (a hard to define category, but one which includes journals which are particularly willing sacrifice publication quality in order to maximise profits) are included in the PubMed Central database (Manca *et al.*, 2018). Pointing to the fact that some article is indexed by PubMed Central as an indication of quality misrepresents the nature of the PubMed Central database and epistemological processes in science more broadly.

## Forms of Non-honesty

In order to facilitate discussion about whether or not the above described communicative tools are honest, we will now introduce some forms of influence which would generally be described as inconsistent with acting honestly (and which might further qualify as dishonest). The descriptions will be necessarily brief and somewhat abstracted, but should help to provide some additional language with which to describe failures of honesty, and identify some of the key features of influence that can undermine honesty.

### Deception

Deception is generally understood to involve intentionally bringing about a false belief in some agent. Thus, whilst one may inadvertently mislead another, one always deceives another on purpose. As with lying, discussed below, there are subtleties which may render this basic sense of deception inaccurate, although it captures the majority of cases of deception. For instance, deception can involve maintaining a false belief in someone who would otherwise have discarded that false belief (Carson, 2010), or it might involve preventing someone from acquiring a true belief that they would otherwise have acquired (maintaining their ignorance). One agent can deceive another without making any utterances. For instance, Jude might deceive Jessica into thinking there is no chocolate left in the house by hiding the last chocolate bar in his room. Misleading people through one’s overt actions (or through omissions) other than utterances could be an important means of deception. Note that one might mislead without deceiving, if one brings about a false belief in another, but does so unintentionally.

There are a number of ways one agent may deceive another. Two of these are through lying and paltering:

#### Lying

Perhaps the most familiar form of deception is lying. The traditional definition of lying is:

*A* lies to *B* if and only if there is a proposition *p* such that

L1. *A* says that *p* to *B*, and

L2. *A* believes that *p* is false, and

L3. By saying *p* to *B*, *A* intends to deceive *B* into believing that *p.* (Stokke, 2013)

There may be exceptions to these conditions – for instance, ‘bald faced lies’ seem to count as lies, but do not require the liar to intend to deceive (since bald faced lies are those told where there is no prospect they will be believed) (Stokke, 2013).^5^ However, paradigmatic lies seem to take the form stated above. Since lying is intentional, it is a form of deception.^6^

Important varieties of lie are the *white lie*, the *noble lie* and the *altruistic lie*. White lies are intended to protect social relations and are often thought benign because they are not of great consequence, or because they are motivated by compassion or similarly benevolent attitudes. Noble lies are lies told – supposedly – in the service of some greater good. Beginning with Plato, the noble lie was a kind of myth that served to reconcile different sectors of society to performing particular roles, functioning to keep people satisfied with their lot and thus obedient (Dombrowski, 1997). In this sense noble lies have been seen by some as part of a totalitarian state and utterly contemptible. In contemporary political discourse, however, the noble lie is commonly used to describe any lie told to the public in order to preserve the stability of the state, and it is unclear whether the paternalism involved in the telling of noble lies is invariably objectionable. Altruistic lies involve A making an untruthful statement to B with the assumption that B will not believe her, and will instead come to believe the truth. Thus, altruistic lies are intended to bring about a true belief via an untruthful statement. On the definition above, they are thus not lies at all, though some, such as Fallis (2009) adopt a definition of lying on which altruistic lies *do* count as lies.^7^

#### Paltering

Perhaps the opposite of an altruistic lie is paltering, or false implicature, which involves bringing about a false belief via a truthful statement (Rogers *et al.*, 2017). To borrow an example from Rogers *et al.* (2017), someone selling a car that has broken down frequently in the past year might, when asked by a potential buyer ‘has this car had any mechanical problems in the past year?’ respond truthfully ‘it drives very smoothly and handles well’. Though true, the statement is intended to mislead. By avoiding misleading via an active lie, the seller may be able to maintain a more positive self-image, or defend herself from accusations of foul play. Paltering is done intentionally, and is thus a form of deception.

### Manipulating

Another behaviour that might appropriately sit under the heading of ‘non-honest’ is manipulation. Many definitions of manipulation exist, some of which stipulate that manipulation is *pro tanto* wrongful, and others which have no such requirement. Manipulation is often taken to involve exerting an influence over an agent’s behaviour by means of non-rational processes, and on this basis manipulation is sometimes taken to include tools such as ‘nudges’ and emotionally laden advertising (Coons and Weber, 2014).^8^ It is unlikely that influence via non-rational means can be cast as *pro tanto* wrongful, at least to the extent that ‘non-rational’ influence is taken to mean influence that does not encourage deliberation and reflection about one’s actions, since such influence is so commonplace. Norms of interaction might dictate when it is inappropriate to utilise non-rational influence, as might factors that affect how valuable it will be to engage in deliberation regarding a particular decision. Manipulation is often contrasted with persuasion and coercion whereby persuasion is the use of rational argument and reason in order to influence behaviour, and coercion the removal of choice in order to control an agent’s behaviour (for discussion see (Blumenthal-Barby, 2012)).

There are many ways of being manipulative. The reason manipulation seems appropriate to include under a discussion of honesty is not that it is generally or invariably associated with the formation of false beliefs about the matter at hand (in contrast to deception). Rather, it is because manipulative influence is often (though not necessarily) covert. It is the aspect of manipulation that gives the *impression* of engaging the subject in rational deliberation, whilst in fact seeking to bypass or undermine those rational deliberative capacities, that makes (some forms of) manipulation particularly troubling from an honesty perspective.^9^

### Bullshitting

An agent who bullshits stops short of lying, but misrepresents themselves (specifically their state of knowledge) to another agent (Frankfurt, 2009). The bullshitter displays an indifference to the truth – they make claims to knowledge which they lack. As such, whilst they may or may not mislead in the sense that what they say may or may not be true (and may or may not be believed), bullshitting always involves an intention to mislead in the sense that the bullshitter gives the impression of believing something to be true, when in fact they have no idea (nor care) whether or not it is true.

### Wishful Speaking

Wishful speaking has been articulated in the context of scientific communication. Wishful speaking is related to, and often mistaken for, the more familiar concept of wishful thinking. Wishful thinking involves an agent *believing* a claim for which she lacks sufficient evidence, motivated by the non-epistemic benefits of believing that claim. In contrast, wishful speaking involves an agent *asserting* a claim for which she lacks sufficient evidence, motivated by the non-epistemic benefits that arise from others believing that claim (John, 2019).^10^ One might claim, for instance, that aromatherapy reduces the pain of childbirth whilst lacking sufficient evidence for this claim, because one will benefit from selling aromatherapy products to expectant parents. Wishful speaking involves invoking an inappropriately low evidential standard for making claims. Depending on the truth value of these weakly-evidenced claims, the agent may mislead or deceive. Wishful speaking might count as an instance of lying, if the speaker does not believe the claim that she makes is true; it might, alternatively, be an instance of bullshitting if she is ignorant – and unconcerned – about its truth status. An agent who is engaged in wishful speaking might also be engaged in wishful thinking (that is, she might believe the claims for which she lacks sufficient evidence, because of the non-epistemic benefits of believing them). Since we know that people engage in motivated reasoning and sometimes adopt beliefs on the basis of their social benefits (rather than epistemic superiority) it would be unsurprising if at least some of those engaged in public health promotion occasionally slip into hopeful (rather than well-evidenced) beliefs and claims about the health benefits of certain behaviours (McKay and Dennett, 2009; Mercier, 2020; Levy, 2021).

## Are Such Communicative Practices Honest?

Our aim, now, is to draw out the ways in which common public health communicative tools share features of the non-honest forms of influence described above. We seek only to highlight the *risk* of non-honesty here, rather than to establish that such practices are *necessarily or invariably* non-honest. In order to make such a judgement, at least for borderline cases, a careful consideration of contextual facts (such as the particular communicative norms in operation, the motivation of the communicator, expectations held by recipients of the goals of the communicator, and so on) is needed. Our concern is that many communicative tools are so familiar and commonly used that the degree to which they ‘distort the facts’ in the service of promoting health goes unnoticed. Since our aim is primarily to draw attention and encourage reflection, rather than to explicitly criticise as ethically unacceptable, we have sought to highlight numerous tools which sit in the ‘grey area’ regarding honesty, rather than to focus on robustly establishing dishonesty in a smaller set of instances.

The practices we have described risk misleading people, in that they might lead to false beliefs at least some of the time. As discussed, such misleading will count as deceptive where it is done intentionally. It is often difficult to definitively judge another agent’s beliefs and intentions, and thus to establish deception. It is likely to be even harder to know the beliefs and intentions held by group agents such as public health institutions, who in many cases will be responsible for public health communications (see (Brown Under Review)). Yet we might look for evidence of intention to mislead. Take, for instance, the statement by the WHO that ‘Breastfeeding improves IQ […]’ (WHO, 2021). As discussed (see *Causation laundering*) evidence for a causal link between breastfeeding and IQ is weak, and it is not unreasonable to expect the authors of the WHO page on Infant and young child feeding to be well aware of the lack of robust evidence to support this causal claim. A disconnect between the evidence and the claims of public health communicators is not conclusive, but is *suggestive* of an intention to mislead, particularly when there are obvious reasons for doing so (in this instance, the perceived need to promote breastfeeding in the face of cultural barriers to doing so). Consider, further, failures to provide people with information about harms alongside benefits; presenting harm and benefit information in mismatched ways (that exaggerate benefits and minimise harms); failing to acknowledge scientific uncertainty; implying causation from correlation, and so on. These are all predictably likely to create inaccurate and overly optimistic beliefs, such that we might expect communicators to foresee (and intended) the creation of false beliefs (Stegenga, 2018; Brown *et al.*, 2022).^11^

It is plausible that, in some cases, public health communicators intentionally present fact-distorting information, with the aim of producing more accurate beliefs downstream (i.e. altruistic lies). Public health communicators concerned that the public will misinterpret factually accurate information might be tempted to ‘tweak’ such information in order to avoid such misinterpretations. Take, for example, evidence that teetotallers suffer worse health outcomes than those who drink a small amount of alcohol. A likely explanation for this finding is that teetotallers are an unusual group, and there may be other reasons why they suffer additional disease burden compared to those who drink alcohol occasionally (e.g. they are recovering alcoholics, they suffer from other conditions that are exacerbated by alcohol). One might be tempted to simplify the story to ‘any alcohol use is associated with some short-term and long-term health risks’ (WHO, 2022) so as to avoid the (presumably incorrect) interpretation that a small amount of alcohol is better for you than becoming teetotal. Yet, whether or not justified, such an altruistic lie should probably not count as honest: it is an intentional fact distortion, albeit with the ultimate goal of making some other beliefs more accurate.^12^ The trade-off between honesty and successful health promotion may sometimes need to be considered, and as we have acknowledge, we do not claim here that honesty must be pursued at all costs. Nor should all genuine, well-intentioned efforts to promote health qualify as honest.^13^

Occasionally public health officials acknowledge their non-honest behaviour. In an interview, Anthony Fauci suggested that he adapted his claims about the percentage of the population that would need to be vaccinated in order to achieve herd immunity, based on his perception of public attitudes towards vaccination (McNeil Jr., 2020; Prasad, 2020; Tufekci, 2020). Rather than communicating what he ‘really thought’, he communicated what he thought people were ‘ready to hear’. He also acknowledged that statements on community mask use were primarily intended to protect supply for healthcare workers, rather than to accurately convey the likely benefit people would receive from wearing a mask when uninfected.^14^ These look like noble lies: intentionally misleading claims made in order to promote the greater good (i.e. achieve greater infection control). As before, arguing that such lies are all-things-considered impermissible is beyond the scope of this paper, but they should at least be recognised as failures of honesty.

Accepting, then, that judgements of intention will rarely be certain let us consider some other forms of non-honest communication. Recall that paltering involves intentionally misleading people via true statements. The use of relative risk to describe the effects of health promoting interventions, the combined use of relative risk for reporting benefits and absolute measures for reporting harms (i.e. mismatched reporting), and the provision of only upper or lower bounds when reporting effects sizes, seem likely to mislead people about the significance of the benefits and harms of interventions, even though they may all be an ‘accurate’ representation of the available evidence.

One hesitation for thinking these practices intentionally inflate expectations, and thus count as paltering, is that the tendency to present effect sizes in terms of relative risks, and the use of mismatched reporting, is present in journal articles and guidelines. For instance, articles published in the *BMJ, The Lancet* and *JAMA* used mismatched reporting when describing the benefits and harms of therapeutic interventions a third of the time, with relative risks typically being used to describe benefits and absolute risks to describe harms (Sedrakyan and Shih, 2007; Wegwarth and Gigerenzer, 2011; Caverly *et al.*, 2016). Public health communicators could thus be copying the presentation of risk information from these sources, rather than intentionally presenting a skewed picture. Another possibility is that communicators think that such a skewed presentation of benefits and harms is likely to lead to a more accurate understanding (i.e. they pursue epistemic paternalism by using misleading information to create true(er) beliefs; an altruistic lie).

Paltering can arise when communicators do not abide by pragmatic principles of cooperative communication. For instance, Powell and colleagues (2018) illustrate how minimally relevant information can have counterproductive effects on knowledge. They explored the impact of the American Diabetes Association’s ‘Diabetes Myths’ website. The authors describe how calling something a ‘myth’ indicates that it is clearly false, whereas ‘the ADA’s “myths” are false only because of some technicality or uncharitable reading’. (Powell *et al.*, 2018). After being exposed to the diabetes myths, participants’ basic knowledge of diabetes went down, rather than up. As discussed by Powell *et al.*, communication requires cooperation and communicators are expected to abide by pragmatic principles, including the principle of relevance (Grice, 1975): if information is provided, the recipient reasonably assumes that that information is relevant. Describing things like ‘People with diabetes can’t eat sweets or chocolate’ and ‘People with diabetes should eat special diabetic foods’ as ‘myths’ implies that they are robustly and relevantly false. Providing minimally relevant or irrelevant information risks causing people to form beliefs that are less accurate than those they previously held.

Manipulation – influence via non-rational means – also seems likely to be present in public health communication. A concern for honesty leads to a worry about manipulative influence that presents information as relevant when it is not relevant, or which disguises the communicator’s beliefs and intentions in some way. One can find health promotion materials that make use of emotive language and imagery without too much trouble, such as anti-smoking posters depicting someone with a fishhook through their mouth, or a child suffocating within a cigarette smoke ‘bag’ (Solopress, 2012), though it is not clear these count as *non-honest* manipulation. Some of the commonplace persuasive language used to encourage people to attend screening (‘life-saving’) or take up exercise (‘miracle cure’) might veer into non-honest manipulation, as might the asymmetrical reporting of harms and benefits, the provision of quantitative data with no or minimal relevance (including denominator neglect), and outcome swamping. Such practices encourage people to neglect important information (such as one’s own risk of death from a particular disease) and instead attend to irrelevant information (such as the global deaths from a particular disease). Since attending to irrelevant information and neglecting relevant information is likely not rational, practices which encourage this should be considered manipulative in a way that is inconsistent with honesty.

Specifying what counts as rational (and non-rational), and hence what counts as manipulation, is controversial. For instance, the tendency to respond to framing effects (such as gain and loss frames) is typically described as a cognitive ‘bias’, yet Gigerenzer (2015, 2021) and others have argued that some such biases are ‘ecologically rational’, since they act as useful heuristics which lead people to make good decisions in real-world settings. Gigerenzer suggests, for example, that the choice of one’s physician to highlight the benefits of an intervention rather than its harms might reasonably be interpreted as intentionally recommending that intervention. We do not wish to suggest that public health communicators should not make recommendations. Yet, if non-honesty is to be avoided, communicators would need to avoid straying across the line between adopting pragmatic forms of communication that are consistent with norms of cooperative communication and exploiting cognitive biases to manipulate people’s behaviour. This could include, for instance, providing all and only relevant information (which may mean including base rate information where relative risks are reported, or avoiding correcting ‘myths’ that are only untrue in a technical, uncharitable sense).

Finally, let us consider bullshitting and wishful speaking, two related practices which sacrifice epistemic ends in order to pursue non-epistemic goals. Both bullshitters and wishful speakers are unconcerned (or insufficiently concerned) with bringing about true beliefs in the recipients of information they provide, and instead makes statements for non-epistemic reasons (such as, for instance, promoting public health through behaviour change).

The practices of offering spurious precision, uncertainty laundering and causation laundering seem likely to involve some combination of bullshitting and wishful speaking. Much epidemiological information is uncertain: it is hard to identify robust causal relationships between health-related behaviours and the various diseases they are thought to cultivate or ameliorate. Yet this uncertainty is rarely communicated to recipients of public health information, giving the impression of more confidence and precision than is warranted. The lack of qualifying uncertainty information in public health communication misrepresents the state of knowledge – specifically, the confidence that claims about the effectiveness of interventions are accurate. Misrepresenting one’s epistemic status in this way is indicative of bullshitting. If, further, the aim of this misrepresentation is to get people to change their behaviour in health promoting ways, then such practices would qualify as wishful speaking. Bullshitters need not be reckless. That is, they might only bullshit about claims that it is hard to prove one way or another, protecting them from being shown to be wrong. Once again, diagnosing bullshitting or wishful speaking may be tricky, and requires assumptions about the intentions of the communicator. Although such diagnoses are speculative, that does not mean they are outlandish.

### Non-honest Behaviour

Our discussion has focused on verbal communication, yet public health officials might sometimes communicate in primarily non-verbal, non-written ways. Dishonest and non-honest behaviour are common in nature. For instance, some birds feign having a broken wing in order to appear vulnerable and draw predators away from their nest. Humans, too, use their overt, non-verbal behaviour as a communicative tool. An infamous example in the public health context was the then Minister for Agriculture, John Gummer’s attempt to feed his four year old daughter a beef burger to reassure the British public during the Bovine Spongiform Encephalopathy (BSE) crisis (BBC, 1990). Assuming Gummer believed British beef to be safe to eat, such performative reassurance was consistent with acting honestly. If, however, Gummer’s behaviour was intended to ‘distort the facts as he saw them’ – if he actually thought it was quite risky to eat British beef (but was willing to expose his daughter to that risk as a ploy to convince the public it was safe) – then such behaviour can be viewed as dishonest.

Behavioural non-honesty might have a different flavour to verbal or written versions. One form of behavioural non-honesty involves the communicator behaving in ways that appear to indicate that they hold a particular set of beliefs (e.g. British beef is safe), when, in fact, they do not holds those beliefs. In this case the communicator performs some behaviour *in order to* communicate to the audience that the world is a certain way, and that they (the communicator) believe the world to be a certain way. In doing so, the communicator intends the audience to come to have a false belief about the world. Other forms of behavioural non-honesty exist (such as stealing), though are less relevant to the public health communication context.

We haven’t discussed non-verbal non-honesty at length here, though we think it could be another important way in which actors involved in public health communication can intentionally mislead audiences, and one worthy of further exploration.

## Concluding Remarks

In this paper, we have considered whether public health communication lives up to the requirements of honesty. Honesty, at its core, involves not distorting the facts as the agent seems them. It is not clear whether public health communication must always be honest. There could be instances where grave threats to public health would justify non-honest or dishonest communication. But the kinds of public health communication that we have considered here do not typically involve such extreme cases. Rather, we have focused on everyday examples of health promotion: encouraging people to lose weight, eat a healthy diet, attend screening programmes, and so on. It seems likely that such communicative practices should be honest, both to ensure people are able to make informed decisions about their health and lives, and to avoid the damage to the reputation of healthcare systems (and subsequent loss of trust) if non-honesty were revealed.

We have suggested that some common public health communicative practices share features with non-honest behaviours. In particular, we suggest that communicators engage in paltering, wishful speaking and bullshitting. Moreover, some communicative practices have the necessary features to count as lies, assuming it can be convincingly argued that communicators *intend* to mislead through their statements. This is, clearly, not uncontroversial. Perhaps the most striking example of (likely) non-honesty in public health communication is the failure to provide information regarding the benefits and harms of health promotion interventions in a transparent and informative format. This includes the decision to provide no quantified effect size estimates much of the time, and the decision to use relative risk and mismatched reporting when quantified estimates are provided; practices likely to inflate expectations of benefit.

Defences of these practices might argue either that they *are* in fact honest, or alternatively, that they are justified, despite being non-honest. The former approach could involve revising the definition we have adopted (‘reliably not intentionally distorting the facts as the agent sees them’), or arguing that altruistic lies, for instance, do not involve fact distortion (since they aim, ultimately, at creating true beliefs). There will be scope for this approach when it comes to a number of the communicative practices we have discussed – defenders often raise the issue of cognitive biases, and the extent to which people misinterpret statistical information. Thus, leaving out quantified risk information altogether, or presenting it in ways that (supposedly) encourage more rather than less accurate risk beliefs, might be defended as fact–*preserving* rather than distorting.

We do not pretend that communicating in an honest way is always easy (particularly whilst also aiming to promote health), nor that judging the honesty of communication will always be straightforward. We would argue that mismatched reporting, even if done with genuine intentions, is likely to mislead and should be considered non-honest. There is an extensive literature on risk communication that describes how (numerical, risk) information can be presented in ways which avoid distortion and support people’s understanding (Wolf, 2012; Bruine de Bruin and Bostrom, 2013; Blastland *et al.*, 2020), including the use of diagrams and fact boxes (Brick *et al.*, 2020). This research does not recommend leaving out numerical information nor ‘correcting’ for biases using skewed risk formatting.^15^

Some of the practices we describe might be defensible, although not fully honest. For instance, the use of spurious precision might, as John (2022) argues, be a case of ‘well-leading’ rather than misleading. That is, recipients of spuriously precise messages, under certain contexts, become more accurately informed as a result, in a manner similar to altruistic lies. We have not discussed whether such cases of ‘epistemic paternalism’ justify non-honest communicative practice. But it would be eccentric to insist that honesty be maintained at all cost, and thus one must consider under what circumstances dishonesty or non-honesty is permissible.

Our hope, in undertaking this discussion, is to highlight how public health communicative practices can fall short of honesty in a variety of ways, and encourage reflection upon whether or not this is appropriate. Ultimately, we hope such reflection would lead to more honest, useful public health communication. Quality kite marks such as the ‘HON Code’ and ‘PIF TICK’ exist and supposedly indicate reliable trustworthy information (Health on the Net, 2020; PIF TICK, 2021). Yet many of the sources we looked at are certified by these external quality markers and yet, we suggest, fall short of honest communication.

In clinical medicine, communicators are required to provide information of material relevance to decisions about how to behave. Distorting information – even in the service of attempting to encourage ‘better’ decisions – is unacceptable. Whilst we have not sought to argue for it here, we suspect the standards for public health communication should be closer to those of clinical medicine. We suspect that, at least some of the time, the reason for the use of non-honest communicative practices is a failure to really appreciate that such practices are non-honest (and the passive acceptance and reproduction of their use). We do not intend to imply that non-honesty is typically undertaken for objectionable or selfish motivations (for instance, financial gain). Instead, we think it likely that the vast majority of non-honest public health communication is benevolently motivated, involving a genuine attempt to effectively promote public health. We only wish for this goal to be balanced with the need to treat people respectfully and avoid employing non-honest practices.

## Footnotes

1 What will count as an honest motivation is up for debate. Miller suggests there are a range of motivations consistent with honesty, but rules out, for instance, motivations that are primarily of self-interest (e.g. an agent who is honest only because she thinks there is a risk that dishonesty will result in censure). On the other hand, agents who recognise that respecting their interlocutor requires honesty, and are thus motivated to behave in honest ways, will qualify as acting honestly.

2 Strictly, the facts ‘as the agent sees them’. Judging how agents – including group agents like public health bodies – see the facts is not straightforward. Yet we can make certain basic assumptions based on the evidence available to public health bodies, expert consensus and so on.

3 Note we have not cherry picked this review: a Cochrane review also concludes that the effects of exercise are uncertain in high quality studies (Cooney *et al.* 2013).

4 We don’t have space to discuss Gricean norms in any depth here. The insight Grice offers, however, is that, since communication is a cooperative exercise, standard norms of communication will dictate the expectations of how speakers and recipients will interpret communications. He offers some maxims of communication which represent standard assumptions/ rules by which communication operates.

5
Lackey (2013) argues that lying always involves a deceptive intention, even if the liar does not intend to bring about a false belief in the recipient.

6 Or at leases involves deceptiveness in some way – see previous footnote: whilst some authors have argued that lies are not always deceptive, Lackey persuasively argues that they always involve a deceptive intention.

7 This definition of altruistic lies is narrower than is sometimes used. Often, altruistic lies are taken to be straightforwardly misleading, though told with benevolent intentions. This is closer to what we have described as a white lie.

8 Note that it is contested whether such influences really are ‘non-rational’ (see, for example, Levy, 2021). Further, it has been directly argued that manipulation need not involve non-rational influence (Gorin 2014).

9 (Much) more has been written on the topic of manipulation, and the potential problems with manipulative influence in the health domain. See, for example, Buss (2005); Rudinow (1978); Blumenthal-Barby and Burroughs (2012); Nys and Engelen (2017); Wilkinson (2013); MacKay (2017).

10 See also related discussion of socially adaptive belief formation (Williams, 2021).

11 There is evidence that patients and doctors both tend to overestimate the benefit and underestimate the harm of clinical medical interventions (Hoffmann and Del Mar, 2015, 2017). The communicative practices discussed above are likely to support the same overestimation relating to public health interventions.

12 This case is close to the grey area where norms of communication/ communicative intentions/ narrow readings of statements might conflict. If I know *for certain* that you always interpret ‘opaque’ to mean transparent and ‘transparent’ to mean opaque, and I tell you the fluid is transparent in order to communicate to you that you cannot see through the fluid, then this does not look like ‘intentional fact distortion’. The WHO alcohol case looks different: the judgement that people cannot interpret alcohol information ‘correctly’ is far more speculative, and given the range of recipients of the communication, surely incorrect for some proportion. To us, this seems like a case of non-honesty (although perhaps not a case of dishonesty).

13 The question of when such altruistic lies are permissible falls outside the scope of this paper. One might imagine altruistic lies told to children to be more frequently permissible than those told to adults, or perhaps altruistic lies from a close friend more permissible than lies from a healthcare provider. In the context of public health communication it is likely to be harder to justify altruistic lies premised on assumptions about, for instance, the influence of cognitive biases on risk processing at the individual and population level.

14 There is some debate over the correct interpretation of Fauci’s statements. The transcript/ original video of the interview can be accessed through the references provided.

15
Zikmund-Fisher (2013) argues that it is necessary to tailor risk formats to user’s needs, for instance, providing simplified formats when users need only order risks in terms of relative magnitude. This may sound reasonable, but in the context of recommending effortful health behaviour changes (which individuals must trade off against numerous other things they value), failing to provide absolute magnitudes hobbles people’s ability to judge the all-things-considered value of such changes. Offering such additional information, where possible (and in the context of online information sources, it is relatively easy to provide additional, nested information) may be of use.
